# Nanomicroarray and Multiplex Next-Generation Sequencing for Simultaneous Identification and Characterization of Influenza Viruses

**DOI:** 10.3201/eid2103.141169

**Published:** 2015-03

**Authors:** Jiangqin Zhao, Viswanath Ragupathy, Jikun Liu, Xue Wang, Sai Vikram Vemula, Haja Sittana El Mubarak, Zhiping Ye, Marie L. Landry, Indira Hewlett

**Affiliations:** Food and Drug Administration, Silver Spring, Maryland, USA (J. Zhao, V. Ragupathy, J. Liu, X. Wang, S.V. Vemula, H.S. El Mubarak, Z. Ye, I. Hewlett);; Yale University School of Medicine, New Haven, Connecticut, USA (M.L. Landry)

**Keywords:** influenza, influenza A, nanomicroarray, next-generation sequencing, viruses, influenza virus, RNA, DNA, genes, hemagglutinin, neuraminidase, subtype

## Abstract

Conventional methods for detection and discrimination of influenza viruses are time consuming and labor intensive. We developed a diagnostic platform for simultaneous identification and characterization of influenza viruses that uses a combination of nanomicroarray for screening and multiplex next-generation sequencing (NGS) assays for laboratory confirmation. The nanomicroarray was developed to target hemagglutinin, neuraminidase, and matrix genes to identify influenza A and B viruses. PCR amplicons synthesized by using an adapted universal primer for all 8 gene segments of 9 influenza A subtypes were detected in the nanomicroarray and confirmed by the NGS assays. This platform can simultaneously detect and differentiate multiple influenza A subtypes in a single sample. Use of these methods as part of a new diagnostic algorithm for detection and confirmation of influenza infections may provide ongoing public health benefits by assisting with future epidemiologic studies and improving preparedness for potential influenza pandemics.

## Abstract 2

This novel platform can detect and differentiate different influenza subtypes from a single sample.

Influenza A virus consists of 8 negative, single-stranded RNA segments encoding 11 proteins: polymerase basic 1 and 2 (PB1 and PB2); polymerase acidic (PA); hemagglutinin (HA); nucleoprotein (NP); neuraminidase (NA); matrix (M1/2); and nonstructural (NS1/2). Influenza A viruses are classified into 18 HA subtypes (H1–H18) and 11 NA subtypes (N1–N11), determined on the basis of the antigenic differences in the surface glycoproteins HA and NA (*1*–*4*). All known HA subtypes of influenza A virus are found in aquatic birds, and some, including H1, H2, H3, H5, H7, and H9, have been reported to infect humans (*1*,*5*–*7*). Direct transmission of avian influenza A virus subtypes H5N1, H7N2, H7N3, H7N7, H9N2, and H10N7 from domestic poultry to humans has been reported (*8*–*13*). 

In early 2009, a novel swine-origin virus, designated influenza A(H1N1)pdm09 (pH1N1), emerged in Mexico and spread rapidly around the world, causing a global influenza pandemic (*14*,*15*). This virus was generated by multiple reassortment events over 10 years (*16*,*17*) and continued to circulate in humans after the initial pandemic period, replacing the previously circulating seasonal H1N1 viruses. Influenza A(H3N2) variant virus (H3N2v) isolated from humans in the United States in 2011 was also generated through reassortment originating from swine, avian, and human viruses, including the M gene from pH1N1 virus (*18*,*19*). More recently, a novel avian-origin influenza A(H7N9) virus capable of poultry-to-human transmission was identified in China (*7**;* http://www.who.int/influenza/human_animal_interface/influenza_h7n9/140225_H7N9RA_for_web_20140306FM.pdf). Diagnosis of infection with this virus is difficult because infection does not kill infected poultry, but the virus may post a substantial risk for a human pandemic because of a lack of immunity in the general population (*7*). As these viruses demonstrate, reassortment of pH1N1 virus with other circulating seasonal strains can produce virulent variants that can be transmitted to and among humans and that could emerge as a future pandemic strain (*15*,*20*,*21*). Therefore, it is critical to determine whether transmitted viruses have pandemic potential in humans during the influenza season. 

Multiple influenza strains are usually prevalent during an influenza season. Increasing global travel results in rapid spread of novel influenza viruses from one geographic region to another (*13*,*22*). Current approaches for screening and characterizing novel influenza viruses require many steps and multiple assays. A single test has not been available for simultaneous identification of newly emerging strains from known or unknown subtypes of influenza viruses and the characterization of unique virulence factors or putative antiviral resistance markers.

We previously described a method for detection of avian influenza A(H5N1) and swine-origin pH1N1 viruses that used a nanotechnology-based, PCR-free, whole-genome microarray assay (nanomicroarray) (*23*,*24*). In this article, we describe a new diagnostic platform for identification and characterization of subtypes of influenza A virus that uses nanomicroarray for screening and multiplex next-generation sequencing (NGS) for laboratory confirmation. We demonstrate that this platform enables accurate and simultaneous identification of multiple subtypes in a single sample. We used this platform to evaluate clinical nasopharyngeal swab specimens from patients with influenza-like illness that had tested positive for influenza virus to determine influenza virus subtype. 

## Materials and Methods

### Oligonucleotide Design and Nanomicroarray Assay

The sequences for multiple capture and intermediate oligonucleotides per target gene were designed and prepared as described previously (*23*,*24*). The oligonucleotide sequences and details of the nanomicroarray assays are listed and described in the Technical Appendix.

### Viruses and Clinical Samples

Information about influenza viruses used in this study is provided in the online Technical Appendix. Nasopharyngeal swab specimens from patients with symptoms of influenza-like illness were submitted to the Clinical Virology Laboratory at Yale–New Haven Hospital, New Haven, Connecticut, USA, during December 27–December 31, 2012. Samples were tested by using direct fluorescent antigen (DFA) test with SimulFluor reagents (Millipore, Billerica, MA, USA) and, in some cases, by real-time reverse transcription PCR (rRT-PCR), as requested by the patients’ physicians. PCR was performed by using the Centers for Disease Control and Prevention rRT-PCR protocol for influenza as previously described (*25*). Samples for which DFA, rRT-PCR, or both gave results positive for influenza A were selected, de-identified, and sent to the Laboratory of Molecular Virology at the Food and Drug Administration in Silver Spring, Maryland, USA, for further testing (Table 1).

**Table 1:** Detection of influenza A viruses in nasopharyngeal swab samples collected from naturally infected patients, Connecticut, USA, 2012–13 influenza season*

Patient ID	Patient age, y/sex	Sample collection			Detection methods			
		Date, 2012	Location		DFA	rRT-PCR, C_t_	Universal PCR	NGS
FLU001	47/F	Dec 30	Hamden, CT		+	ND	+	H3N2
FLU002	80/M	Dec 30	Milford, CT		+	17.5	+	H3N2
FLU004	35/F	Dec 30	Meriden, CT		+	ND	+	H3N2
FLU006	25/M	Dec 29	New Haven, CT		+	ND	+	H3N2
FLU007	23/F	Dec 30	New Haven, CT		+	ND	+	H3N2
FLU008	31/F	Dec 30	Trumbull, CT		+	28.1	+	H3N2
FLU009	68/M	Dec 30	Hamden, CT		+	ND	+	H3N2
FLU012	35/F	Dec 30	Rutledge, MO		+	ND	+	H3N2
FLU013	92/M	Dec 29	Woodbridge, CT		+	16.6	+	H3N2
FLU014	84/F	Dec 30	Chester, CT		+	15.5	+	H3N2
FLU017	66/F	Dec 29	Clinton, CT		+	24.3	+	H3N2
FLU018	17/M	Dec 31	New Haven, CT		+	19.9	+	H3N2
FLU021	63/F	Dec 30	New Haven, CT		+	ND	+	H3N2
FLU023	55/F	Dec 28	North Haven, CT		+	21.8	+	H3N2
FLU025	47/M	Dec 30	West Haven, CT		+	ND	+	H3N2
FLU026	32/F	Dec 28	West Haven, CT		+	ND	+	H3N2
FLU027	26/F	Dec 30	Bridgeport, CT		+	ND	+	H3N2
FLU028	89/F	Dec 29	Woodbridge, CT		I	21.0	+	H3N2
FLU033	82/F	Dec 29	Guilford, CT		+	19.8	+	H3N2
FLU034	37/F	Dec 27	New Haven, CT		+	ND	+	H3N2
FLU036	18/M	Dec 29	New Haven, CT		I	17.8	+	H3N2
FLU037	8/F	Dec 29	New Haven, CT		+	ND	+	H3N2
FLU038	21/M	Dec 27	West Haven, CT		+	ND	+	H3N2
FLU040	23/M	Dec 27	West Haven, CT		+	ND	+	H3N2

*C_t_, cycle threshold value; DFA, direct fluorescent antigen test; I, inadequate cells for DFA; ID, identification; ND, not done; NGS, next-generation sequencing; rRT-PCR, real-time RT-PCR.

### Viral RNA Extraction and rRT-PCR

A previously reported universal primer designed to amplify all 8 gene segments (*26*,*27*) was modified by adding 13-bp flanking sequence (5′-ACGACGGGCGACA-3′) at the 5′ end of each primer to enhance the annealing temperature and achieve high fidelity and yield in PCR amplification. Additional details of RNA extraction and rRT-PCR conditions are described in the Technical Appendix.

### NGS Assay

The concentration of PCR amplicons of all 8 gene segments of influenza A virus was measured by using the Qubit dsDNA BR Assay System (Covaris, Woburn, MA, USA); 1 ng of DNA product was processed for NGS sample preparation by using a Nextera XT DNA Sample Preparation Kit (Illumina, San Diego, CA, USA), according to the manufacturer’s instructions. Briefly, the Nextera XT transposome fragmented PCR amplicons into a size of ≈500–700 bp and added adaptor sequences to the ends, enabling a 12-cycle PCR amplification to append additional unique dual index (i7 and i5) sequences at the end of each fragmented DNA for cluster formation. Mega-amplicons from influenza virus were internally marked with these dual-barcoded primers, which enabled multiplexing and simultaneous detection of different subtypes in the same run. After purification of PCR fragments and library normalization, sample pooling was performed by mixing equal volumes of each normalized DNA library, and the barcoded multiplexed library sequencing was performed on an Illumina MiSeq (Illumina). After automated cluster generation, sequencing was processed and genomic sequence reads obtained.

### Bioinformatics Analysis

Sequencing reads of ≈300 bp were dynamically trimmed and sequence data were verified by FastQC software (http://www.bioinformatics.babraham.ac.uk/projects/fastqc/) before de novo assembly. The genome-contiguous assembly was constructed from MiSeq reads by using a de novo module in CLC genomics workbench software version 6.0.2 (CLC bio, Cambridge, MA, USA); minimum contiguous length was set at 800 for assembling consensus sequences (*28*). A comprehensive-read database was generated for the whole genome of the influenza virus tested. Sequences were further filtered so that the local database contained only 1 unique contig for each gene segment, and multiple contigs were generated for each sample. These representative sequences comprise the set of unique sequences from the dataset. A FASTA file with all unique contiguous sequences of each mega-amplicon was used to perform an all-by-all Identify Similae Sequences search in the Influenza Research Database (IRD, http://www.fludb.org), the Global Initiative on Sharing All Influenza Data database (http://platform.gisaid.org), and the National Center for Biotechnology Information database (http://www.ncbi.nlm.nih.gov). The top-scoring BLAST (http://blast.ncbi.nlm.nih.gov/) match was selected to identify the specific genome. Assembled sequences were aligned in ClustalW (http://www.clustal.org), and phylogenetic analysis was performed in MEGA by using the neighbor-joining method (*29*). All amplicons were accurately categorized into a typical subtype.

## Results

### Verification of Capture and Intermediate Oligonucleotides

A nanomicroarray for each target gene was designed, printed in-house, and tested separately by using the PCR products as templates to verify the ability of individual capture and intermediate oligonucleotides to detect a specific target gene. Ineffective capture oligonucleotides were replaced and retested. We amplified PCR products of HA, NA, and M genes for H7N2, H7N3, and H9N2 viruses separately or simultaneously in a single reaction using the corresponding 3 sets of specific primers. To identify the correct HA and NA gene segments for multiplex influenza subtyping, we fabricated a new nanomicroarray by pooling autologous 4 to 5 capture oligonucleotides for a specific gene and then printing them on the array substrate in triplicates. Each nanomicroarray subarray contains multiple gene spots for multiplex assays. The PCR products were hybridized on the array, and the specific signal profiles were correctly observed in the areas printed with corresponding gene-specific capture oligonucleotides (Technical Appendix Figure 1). No interference or cross-hybridization was observed when multiple targets and intermediate oligonucleotides were included in the assay. The specific signal pattern showed the assay’s ability to accurately discriminate influenza subtypes. 

### Amplification of Whole-Genome Segments

To further confirm subtypes detected in the nanomicroarray screening assay for final laboratory diagnosis, we redesigned universal primers to amplify whole-genome segments and separately tested 22 influenza A strains covering 10 subtypes and 3 influenza B viruses. All 8 segments of influenza A viruses were simultaneously amplified in a single reaction, resulting in multiple PCR products ranging in size from 500 to 2,500 bp (mega-amplicons). We also tested 3 influenza B viruses, B/Brisbane/60/2008 (Victoria lineage), B/Pennsylvania/7/2007 (Yamagata lineage), and B/Victoria/304/2006 (Victoria lineage), and 2 influenza A viruses, A/Panama/2007/1999 (H1N1) and A/ruddy turnstone/NJ/65/1985 (H7N3), and found several faint, nonspecific small bands no larger than 1 kb (data not shown).

### Evaluation of Nanomicroarray Assay by using PCR Mega-amplicons

**Figure 1:**
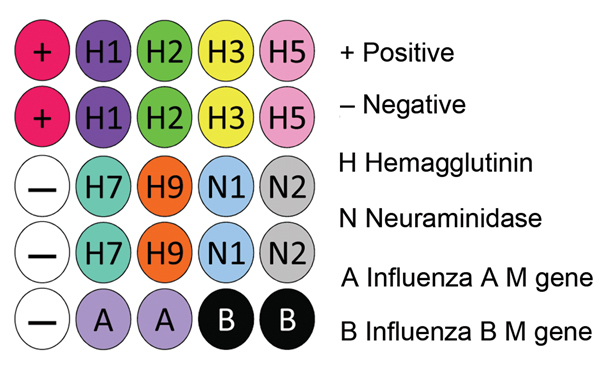
Nanomicroarray layout design for testing of samples for influenza A and B viruses. The microarray internal positive control capture is listed in Technical Appendix Table 1. The negative control is the printing buffer. M, matrix protein.

**Figure 2:**
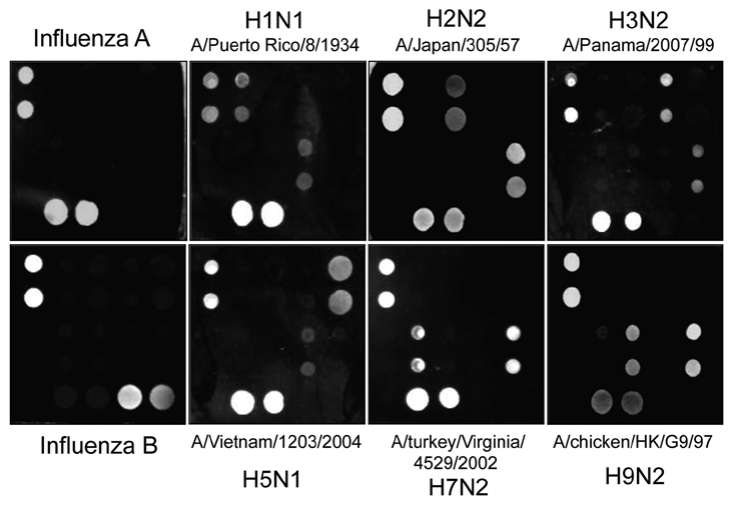
Portion of the microarray images for DNA oligonucleotides of influenza viruses after hybridization with PCR products. Lighter shades represent greater silver intensities for each gene. Typical nanomicroarray silver staining images represent the hits for specific types or subtypes indicated. The positive controls of influenza A and B (left panels) use PCR products amplified by pair-specific primes for matrix gene.

To include M gene capture oligonucleotides for influenza B viruses, we developed a new nanomicroarray (Figure 1). As shown in Figure 2, amplified PCR products of the matrix gene from influenza A and B viruses were specifically detected in the correct spot areas without cross-hybridization. The mega-amplicons of influenza A viruses were correctly identified and found to have a unique fingerprint for each influenza A virus tested. Each spot pattern represented a typical influenza virus subtype corresponding to the gene-specific capture oligonucleotides. We conclude that the current nanomicroarray assay can simultaneously discriminate influenza A from B viruses and specifically identify influenza A subtypes H1, H2, H3, H5, H7, H9, N1, N2, and N3.

### NGS Confirmation of Influenza A Subtypes

A total of 17 mega-amplicons representing 10 subtypes of influenza A and 1 of influenza B were tested in the NGS assay. Multiple contiguous sequences were created automatically for each mega-amplicon by using a de novo assembly program in CLC, and 4–8 contigs supported by high coverage rate of sequence reads were generated (Technical Appendix Table 2). The mega-amplicons of the A/Vietnam/1204/2004 (H5N1) strain yielded 8 contiguous sequences supported by 90,962 reads. Further BLAST search of 8 contiguous sequences in the IRD resulted in 8 mast BLAST reports. All contiguous sequences were found to correspond to 6 proteins (PB2, PB1, PA, NP, M, and NS) of A/Puerto Rico/8/1934 (H1N1) virus and 2 proteins (HA/CIP045/CY077101 and NA/HM006761) of A/Vietnam/1203/2004 (H5N1) with 99%–100% sequence identity. These results showed that all amplicons were correctly identified as the H5N1 laboratory strain. 

The mega-amplicons from A/turkey/Virginia/4529/2002 (H7N2) and A/Minnesota/10/2012 (H3N2) strains resulted in 8 contigs, all correctly identified as the correct subtype. We found strong concordance in contiguous sequences and PCR fragments for each mega-amplicon. A de novo assembly program generated at least 7 contigs from faint band mega-amplicons for influenza B virus (B/Brisbane/60/2008); 2 showed good coverage (7,077 and 10,168), but the BLAST search indicated that none matched the gene sequence from this strain. Further investigation using freshly extracted RNA may be required.

### Simultaneous NGS Discrimination of Multiple Subtypes in a Single Sample

We tested 4 influenza viruses obtained from the Centers for Disease Control and Prevention, A/Puerto Rico/8/1934 (H1N1), A/Vietnam/1203/2004 (H5N1), A/Minnesota/10/2012 (H3N2), and A/Anhui/1/2013 (H7N9), to determine the presence of H1, H3, H5, H7, N1, N2, and N9 subtypes. RNA was extracted from individual or mixed viral strains. The universal rRT-PCR was performed to amplify whole-genome segments in which the PCR mega-amplicons represented a similar pattern to individual or mixed viral samples (Technical Appendix Figure 2). After the NGS assay followed by de novo assembly, 8 contigs were generated for the H5N1 and H3N2 viruses, and 15 contigs were generated for the mixed sample, supported by a high coverage rate of reads (Table 2). These 15 contigs from mixed samples exactly matched the gene segments of the H5N1 and H3N2 strains, similar to the results obtained with the individual sample, as expected. HA and NA genomic sequences of the H7N9 subtype virus were identified as strain A/Anhui/1/2013 from the Global Initiative on Sharing All Influenza Data database. Because PB2 (2,341 bp), PB1 (2,341 bp), and PA (2,233 bp) genes have very similar sizes, direct separation of each gene from co-infected samples is not possible by conventional sequencing methods. These findings demonstrated that the NGS assay can simultaneously identify and confirm the presence of >1 influenza subtypes in a single sample.

**Table 2:** Summary of results from NGS data analysis for influenza A(H3N2) and A(H5N1) viruses obtained from the Centers for Disease Control and Prevention

Strain	NGS total contigs/reads	Findings	Gene segment (length, bp)							
			PB2 (2,341)	PB1 (2,341)	PA (2,233)	HA (1,778)	NP (1,565)	NA (1,413)	M (1,027)	NS (890)
A/Vietnam/1203/ 2004(H5N1)	8/125,438	Length, bp	2,032	1,801	1,841	1,747	1,120	1,259	1,075	894
		Read count	7,604	2,019	18,762	16,925	6,490	24,192	20,127	920
A/Minnesota/10/ 2012(H3N2)	8/162,155	Length, bp	2,251	2,192	2,081	1,754	1,556	1,639	1,171	876
		Read count	30,002	16,392	19,253	15,194	21,818	8,119	25,879	18,519
A/Vietnam/1203/ 2004(H5N1) and A/Minnesota/10/ 2012(H3N2)	15/150,756	Contigs, H5	2	1	13	14	4	6	7	
		Length, bp	2,013	2,115	1,815	1,746	1,476	1,193	936	
		Read count	7,003	6,857	4,359	3,735	854	10,895	15,127	
		Contigs, H3	15	5	10	11	3	12	8	9
		Length, bp	2,530	2,393	1,959	1,465	1,565	1,633	1,103	925
		Read count	15,521	6,753	4,113	7,743	13,808	11,147	5,411	6,110

*de novo assembly module was used in CLC Genomics Workbench software version 6.0.2 (CLC bio, Cambridge, MA, USA) for result handing. Minimum contiguous length was set for 800 to assemble the consensus sequences. NGS, next-generation sequencing; PB, polybasic; PA, polymerase; HA, hemagglutinin; NP, nucleoprotein; NA, neuraminidase; M, matrix; NS, nonstructural.

### Evaluation of Nasopharyngeal Swab Samples by using NGS Assays

We performed universal RT-PCR and NGS assays on 24 nasopharyngeal swab samples obtained from patients who had received a diagnosis of influenza. These samples were initially tested by DFA, rRT-PCR, or both and found to be positive for influenza A virus. After decoding, all samples were found to be positive by using the universal RT-PCR detection method, indicating presence of influenza A infection (Table 1). When mega-amplicons representing the 24 patient samples were tested in the NGS, a total of 32.8 million reads were obtained, and multiple contigs were generated for each sample (Technical Appendix Table 3). A BLAST search of each contig in the IRD database identified the genome corresponding to the influenza A(H3N2) subtype. The coverage of influenza A(H3N2) genomes in the NGS assay was 96.7% (31.7/32.8 million) of raw reads and 76.6% (183/239) of total contigs. A total of 95.3% (183/192 contigs) of the influenza A(H3N2) genome was amplified and sequenced; the average depth of coverage for each contig was 3,259. Of these genomes, 71% (136/192) of segments yielded full-length sequences; HA genes were 96% (23/24); NP, 96% (23/24); NA, 88% (21/24); M, 88%, (21/24); and NS, 79% (19/24). The average breadth of coverage was 100% for HA, NA, NP, M, and NS genes and 93% for PB2, PB1, and PA genes. 

**Figure 3:**
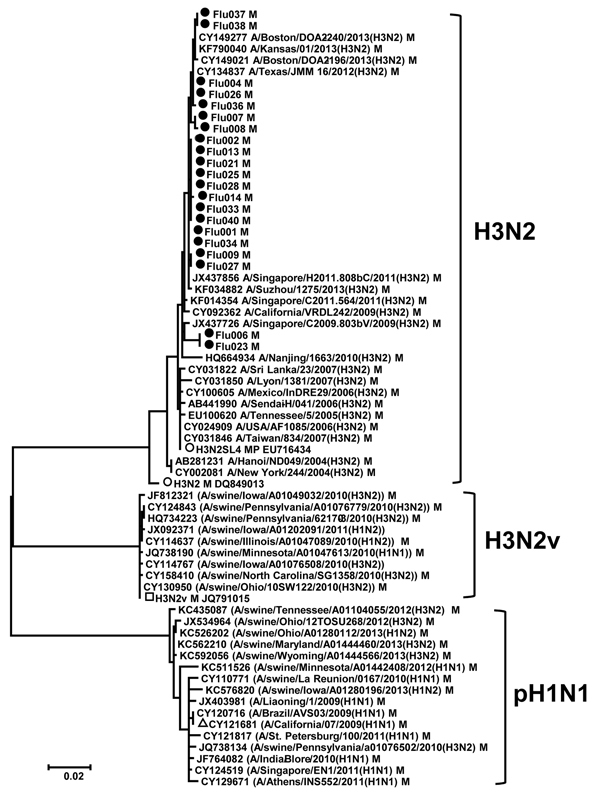
Phylogenetic analysis of the matrix (M) gene sequences obtained from nasopharyngeal swab samples from patients who had received a diagnosis of influenza in Connecticut, USA, during the 2012–13 influenza season (see Table 1). Analysis was performed by using the neighbor-joining module in MEGA (*29*) with the Kimura 2-parameter method. The reference subtypes were fetched from the Influenza Research Database (http://www.fludb.org) and used to construct the tree. Bootstrap values >70% are shown. The M genes identified in this study are indicated by black circles; reference M genes are indicated by black squares for influenza A(H3N2)v and black triangles for pandemic influenza A(H1N1) 2009 (pH1N1) virus. Scale bar indicates 2% genetic distance.

Phylogenetic analysis of each of the 8 segments separately for all isolates showed that all genes clustered together in the H3N2 radiation with a high bootstrap value (data not shown). None of the M genes closely clustered with the M genes from the pH1N1 or H3N2v viruses (Figure 3), which suggests that these viruses are not H3N2v (*18*,*19*). The genotype of 24 influenza A(H3N2) viruses was determined as [A,D,B,3A,A,2A,B,1A] by using FluGenotyping (http://www.flugenome.org), which indicates that the same lineage virus is circulating in this region. The HA genes from most samples shared very high identity with A/Boston/DOA2–206/2013(H3N2) and the NA genes with A/Boston/DOA2-141/2013(H3N2) strain. After completing these analyses, 181 gene sequences were deposited into GenBank (accession nos. KJ741883–KJ742063).

## Discussion

We report the development of a novel diagnostic platform for simultaneous detection, typing, and whole-genome characterization of influenza viruses that uses a combination of nanomicroarray and high-throughput NGS approaches. First, we designed capture and intermediate oligonucleotides for H1, H2, H3, H5, H7, H9, N1, N2, and N3 of influenza A virus and M genes of influenza B virus and evaluated these oligonucleotides in a nanomicroarray assay. Second, we modified previously reported universal primers (*26*,*27*) and used them to amplify the whole genome of influenza A viruses for validation of the nanomicroarray assay. Finally, we confirmed results by using the NGS assay. This protocol enables random accessing of a variety of target genes for simultaneous identification and final sequence-based confirmation of influenza virus infection.

Designing multiple capture and intermediate oligonucleotides with sequences covering the entire genome ensures specific capture of multiple target genes on the nanomicroarray and subsequent detection with a universal nanoparticle probe regardless of mutation, deletion, and influenza reassortment. Furthermore, this design is adaptable for other applications and enables direct detection and subtyping of an unknown sample without previous knowledge of types and subtypes. In the current format, >50 degenerate capture oligonucleotides cover 12 influenza viral target genes, enabling direct detection of any combination of ≈20 subtypes in a single sample, identification of influenza A subtypes in a single assay, and differentiation of influenza A from B viruses. An optimal nanomicroarray assay, which is a reformatted portable device modified for use in point-of-care settings, should include target genes from most influenza A and B viruses as well as for other respiratory viral pathogens. The assay should be easily performed by an untrained technician for sample testing in the field without enzymatic reactions, and results should be in a form that can easily be visualized by the naked eye. In comparison to other conventional detection methods for targeting each gene of influenza A and B viruses separately, the nanomicroarray assay is a one-test-fits-all approach for diagnosis of influenza virus infections that can provide results in <1.5 hours, making this method relatively cost- and time-effective. More important, the nanomicroarray assay can detect emerging and reassortant viruses, and those samples can be sent to centralized laboratories that perform the NGS assay for final sequence confirmation.

Gene segments in most influenza viruses isolated from humans can be adapted from animals, as shown by genetic changes in influenza A(H7N9) isolates from poultry and humans (*7*,*30*). These studies indicated that more changes were acquired during the human infection process. Determining the nature and frequency of co-infection associated with influenza A virus will be critical if an unknown sample contains a novel strain or >1 HA or NA gene subtype. NGS is a powerful tool facilitating diagnosis on a large scale, including high-throughput and simultaneous identification of >96 samples barcoded by using dual index primers and detection of >9,216 genes in a single sequencing run. By using a universal primer adapted to fabricate the mega-amplicons, we showed that the NGS assay is capable of accurately subtyping any influenza A virus and detecting multiple known and unknown influenza genes in a single assay. Bioinformatic skills and mathematics tools, combined with epidemiologic studies, are useful in facilitating prediction of potential subtypes according to the genetic matrix composition of influenza genomic segments for a new, emerging, and reassorted strain whereby the subtypes can be confirmed (*31*–*34*). Influenza A viruses representing 11 subtypes were accurately detected in this study, and the 2 mixed influenza viruses were discriminated by using this sequencing-based diagnostic platform. 

Sequence analysis of 24 clinical samples revealed that 23 (92%) segments contained an amino acid substitution at position E627K in PB2 gene. Mutation of glutamic acid (E) at PB2 residue 627 to lysine (K) favors adaptation to the mammalian host; such mutations have been found in human isolates of highly pathogenic avian influenza viruses of the H7N7 and H7N9 subtypes (*7*,*35*,*36*). These mutations might confer high virulence to the virus by enhancing replication efficiency, increasing polymerase activity and disease severity of avian influenza viruses in mammals (*37*). 

Of the 24 M genes of the samples we tested, 21 (88%) had a single S31N mutation in the transmembrane region of the M2 protein, which has been found to confer resistance to amantadine (*7*,*38*). The emergence of E627K(PB2) and S31N(M2) mutations in tested samples suggests that human host infection in the Connecticut region in the 2012–2013 seasons might be poultry-to-human transmission associated with disease severity (*39*,*40*). This observation highlights an increased risk to public health and the need to continually monitor isolates obtained from mammal reservoirs for genetic variation. This information may help guide clinical treatment and assessment of epidemiology during the epidemic season.

The assay we evaluated is a minimally manipulated procedure that greatly reduces the number of amplifications and omits fragment separation and purification. It is therefore suitable for identification of any strains of influenza virus. An ongoing study using this assay has simultaneously detected and confirmed influenza A(H3N2), pH1N1, and influenza B viruses in >100 nasopharyngeal swab samples (J. Zhao et al., unpub. data). 

**Figure 4:**
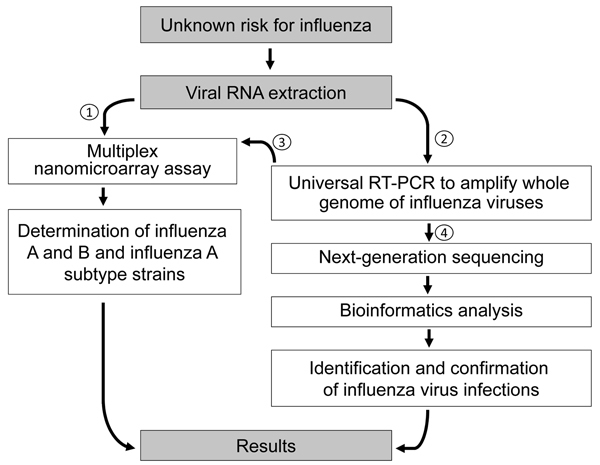
Diagnostic algorithm for identification of an unknown risk for influenza by using nanomicroarray and next-generation sequencing (NGS) assays. To determine the virus type for a suspected influenza virus infection, viral RNA is extracted from a patient sample and initially analyzed in nanomicroarray assay for screening and determining the influenza A and B viruses (1). Once a novel, emerging, or co-infected influenza A and B virus is found, universal reverse transcription PCR (RT-PCR) is performed to generate whole-genome mega-amplicons (2), which can then be retested on the nanomicroarray assay to confirm the initial finding (3) or sent to the central laboratory performing the NGS assay and data analysis for final sequence confirmation (4).

This detection platform provides a new, accurate, and rapid method to refine the differential diagnosis of influenza by selecting a single test or a small set of tests to determine the strain or strains present in a single clinical sample. We propose a new diagnostic algorithm based on this combined platform for identification and characterization of infection risks of unknown influenza strains (Figure 4). For testing a suspected influenza virus infection, this detection platform takes 2–3 days to perform NGS assay and data analysis. However, it provides whole-genome characterization and a final report in matrix type by which a potential pandemic prevalence strain can be predicted, with data including the genetic variant, amino acid signatures for virulence factors, and drug-resistance– and host-adaptation–associated mutations.

Future studies need to be conducted to reformat the current microarray to a point-of-care setting and to expand testing of clinical samples to other geographic regions and additional influenza virus types/subtypes. The NGS assay involves sample preparation and generates massive sequence data for the final report for test interpretation, which requires a higher level of performance for clinical assay validation. Development of an automated assembly and analysis pipeline can make the bioinformatics analysis of transferring raw reads to the specific genomic identification more efficient. This molecular diagnostic platform has the potential for monitoring newly emerging or re-emerging viral reassortants derived from different precursors and could be included as a part of pandemic influenza surveillance strategies for efficient prevention and timely implementation of treatment to protect and improve public health.

Technical Appendix Detailed methods for the design of capture and intermediate oligonucleotides, testing of viral and clinical samples, reverse transcription PCR, and nanomicroarray printing and testing of samples.
