# Supplementary material for: Nanomicroarray and Multiplex Next-Generation Sequencing for Simultaneous Identification and Characterization of Influenza Viruses
Source: Emerg Infect Dis. 2015 Mar;21(3):400–8. doi: 10.3201/eid2103.141169 (PMC4344273; doi:10.3201/eid2103.141169)
Supplement: Technical Appendix — Detailed methods for the design of capture and intermediate oligonucleotides, testing of viral and clinical samples, reverse transcription PCR, and nanomicroarray printing and testing of samples. [file 14-1169-Techapp-s1.pdf]

Article DOI: http://dx.doi.org/10.3201/eid2103.141169

Nanomicroarray and Multiplex Next-Generation Sequencing for Simultaneous Identification and Characterization of Influenza Viruses

Technical Appendix

Design of capture and intermediate oligonucleotides

The sequences for capture and intermediate oligonucleotides were designed and prepared as described previously. In brief, by using nucleotide sequences available in the National Center for Biotechnology Information (NCBI) influenza resource (http://www.ncbi.nlm.nih.gov/genomes/FLU/) and the Influenza Primer Design Resource (IPDR) (http://www.ipdr.mcw.edu/fludb/search), we performed multiple sequence alignments of HA, NA and M genes with MEGA 5 and Vector NTI Advance 11 (Invitrogen, Foster City, CA). Over 120 known sequences of HA and NA genes of different subtypes were selected from ≈500 hit count analyses of each gene covering sequences of viruses from different geographic regions (i.e., Northern America, Europe, and Asia) and different time periods. The selected nucleotide sequences were further aligned to identify significant divergent or conserved regions for detection and differentiation of subtypes. Multiple degenerate capture and intermediate oligonucleotides (usually four or five, respectively) complementary to the conserved regions covering the entire genome of each subtype were designed. This critical design ensures that the target gene is captured on the microarray, hybridized with intermediate oligonucleotides and detected using the gold nanoparticle probe. The designed intermediate oligonucleotides modified with 5′-amino-C_6-modifier while a 25-mer poly (A) tail were added at the 3′ end of intermediate oligonucleotides during synthesis (Integrated DNA Technologies, Coralville, IA, USA). Capture oligonucleotides that did not bind to any known sequence of influenza A viruses were included as array internal positive controls. The oligonucleotides sequences are listed in Technical Appendix Table 1.

Viruses and clinical samples

Influenza virus isolates were propagated in 9–11 day-old embryonated chicken eggs at 33°C for 48h and then aliquoted and stored at −70°C until use in the FDA Center for Biologics Evaluation and Research. Virus infectivity was determined by plaque assay using MDCK cells or 50% egg infectious dose (EID_{50}). Some influenza reference strains were provided by Dr Stephen Lindstrom (Centers for Disease Control and Prevention, Atlanta, GA) and Dr Maryna Eichelberger (Food and Drug Administration, Silver Spring, MD) or purchased from ZeptoMetrix (ZeptoMetrix Corp., Buffalo, NY). Fifteen different strains, A/Puerto Rico/8/1934 (H1N1), A/California/04/2009 (pH1N1), A/Japan/305/1957 (H2N2), A/Panama/2007/1999 (H3N2), A/Brisbane/10/2007 (H3N2), A/Minnesota/10/2012 (H3N2), A/Indiana/08/2011 (H3N2v), A/Vietnam/1203/2004 (H5N1), A/turkey/Virginia/4529/2002 (H7N2), A/ruddy turnstone/NJ/65/1985 (H7N3), A/chicken/Hong Kong/G9/1997 (H9N2), A/Anhui/1/2013 (H7N9), B/Brisbane/60/2008 (Victoria lineage), B/Pennsylvania/7/2007 (Yamagata lineage), and B/Victoria/304/2006 (Victoria lineage), were selected for the nanomicroarray and NGS assays.

Reverse transcription PCR

Viral RNA was extracted directly from allantoic fluid or cell culture supernatants with QIAamp Viral RNA Mini Kit (QIAGEN, Valencia, CA). The purified RNA was quantified using a NanoDrop UV spectrometer (NanoDrop Technologies, Inc., Rockland, DE). Viral RNA was first transcribed into cDNA using a SuperScript III First-Strand Synthesis System for RT-PCR (Invitrogen) according to the manufacturer’s instructions. The cDNA was then used as a template for RT-PCR. To evaluate capture oligonucleotides in the nanomicroarray assay, PCR primers sets for amplification of HA, NA, and M genes of H2N2, H7N2, H7N3 and H9N2 were designed and are listed in Technical Appendix Table 1. The PCR products (size: 1747 bp, 1335 bp, and 1015 bp) of three gene segments were amplified simultaneously for these viral subtypes. In addition, a universal primer set previously reported to amplify all eight gene segments was modified by adding a 13 bp in length of flanking sequence (5′-ACGACGGGCGACA-3′) at the 5′ end of each primer to enhance the annealing temperature and achieve high fidelity and yield in PCR amplification. Reverse transcription (RT) was performed with a uni12 primer SuperScript III First-Strand Synthesis System for RT-PCR. For amplification of all eight gene segments,

PCR was performed in a total volume of 30 µL containing 1 µL of cDNA, 15 µL of 2xPCR buffer (Extensor Hi-Fidelity ReddyMix PCR Master Mix, ABgen House, Surrey, UK), 2.5 pmol of forward and 2.5 pmol of reverse primers (unifluaf and unifluar). Reaction conditions included one cycle at 94°C, 5 min, 35 cycles at 94°C, 30 sec, 50°C, 40 sec, 68°C, 2.4 min, and one cycle at 68°C, 7 min. The PCR products were electrophoresed with 2.0% agarose gel slabs in Tris-acetate-EDTA buffer to observe multiple amplicons. PCR products were quantified using an Agilent 2100 Bioanalyzer (Agilent Technologies, Inc., Wilmington, DE), detected using the nanomicroarray assay and finally sequenced using the NGS assay. PCR was performed in Clinical Virology Laboratory at Yale New Haven Hospital using the CDC real-time reverse transcription PCR (RT-PCR) protocol for influenza (http://www.who.int/csr/resources/publications/swineflu/realtimeptpcr/en/index.html) on an ABI 7500 instrument (Applied Biosystems, Foster City, CA) (*25*).

Nanomicroarray printing and testing of samples

The printing solution contained 50 mM sodium phosphate at pH 8.5, 65% Pronto solution, 0.05% SDS, 0.01% glycerol, and 5 µM capture oligonucleotides. Four to five captures were prepared individually or mixed for identification of a typical gene segment. The nanomicroarray format was designed and the capture oligonucleotide array printed on CodeLink Activated slides in a double- or triple-spot format using an OMNIGrid Accent printer (Genomic Solutions Inc., Marlboroug, MI). Each slide contained 10 identical sub-arrays segregated by a hybridization gasket, thus allowing simultaneous testing of 10 samples per slide. Aqueous DNA-conjugated gold NP-probe and silver staining solutions were purchased from Nanosphere Inc (Northbrook, IL, USA). One µL of 1× to 10× diluted PCR products was used as template in nanomicroarray assay, RNA/DNA samples and 10 nM of the intermediate oligonucleotides were mixed in 100 µL of the hybridization buffer containing 5×SSC, 0.05% sorbitan mono-9-octa decenoate poly(oxy-1,1-ethanediyl), 0.05% Tween-20 and 40% formamide, applied to the nanomicroarray and incubated for 30 min at 40°C with orbital shaking at 500 rpm. PCR amplicons were denatured at 95°C for 5 min and cooled on ice for 2 min before being loaded on to the array. After three washes with wash buffer A (0.5 N $NaNO_3$, 0.01% SDS and 0.05% Tween-20) and one rinse with wash buffer B (0.5 N $NaNO_3$), the universal nanoparticle (NPs) probe was added to the slide and incubated for 30 min at 40°C. Slides were subsequently stained

with the Silver Enhancer A and B solutions for 5 min at room temperature. The light-scattering signal produced by silver-enhanced gold NPs was captured using a photosensor and converted to a TIFF image using a Verigene reader for analysis of the nanomicroarry assay data described previously. The resulting TIFF images were analyzed using GenePix Pro 7 software (Molecular Devices, San Francisco, CA).

Technical Appendix Table 1. Oligonucleotide sequences for capture (c), intermediate (i), and PCR

Oligo ID	Gene	Purpose	Sequences (5′ to 3′)
cFluBM1	Flu B M	capture	TGGGAAAGARTTTGACCTAGACTCTGCYTTGGAATGGATA
cFluBM2	Flu B M	capture	ATTCAATGCAAGTAAAACTAGGAACGCTCTGTGCTTTRTG
cFluBM3	Flu B M	capture	GGAAGGRATTGCAAAGGATGTAATGGAAGTGCTAAAGCAG
cFluBM4	Flu B M	capture	GAGACAATAAACAGAGAGGTATCAATTTTGAGACACAGTT
cH21	H2	capture	RAAYGTCACYGTGACTCAYGCCAAGGACATTCTTGAGAAR
cH22	H2	capture	YTGYAGCATTGCMGGATGGCTCCTTGGAAATCCAGAATGT
cH23	H2	capture	GGTAATYTAATTGCACCAGAGTATGGWTTCAAAATATCGA
cH24	H2	capture	TGTTTGGRGCAATAGCTGGTTTTATAGARGGNGGATGGCA
cH25	H2	capture	CRTATGATTATCCCAARTATGAAGARGAGTCYAARCTRAA
cH71	H7	capture	GACAAAATATGTCTTGGGCACCATGCTGTGGCAAATGGAA
cH72	H7	capture	GACAARATWTGYCTTGGGCAYCATGCYGTGSCAAAYGGRA
cH73	H7	capture	GTTCTTCTTTCTATGCAGAGATGAAGTGGTTGCTGTCGAA
cH74	H7	capture	GWTCYTCWTTCTATGCRGARATGAARTGGYTDYTGTCRAA
cH75	H7	capture	TTCACTTTCAATGGGGCATTCATAGCCCCTGACAGGGCAA
cH76	H7	capture	TTCASYTTCAATGGGGCATTCATAGCYCCWGAYMGDGYAA
cH77	H7	capture	TGGGAGGGTCTCATCAATGGATGGTATGGTTTCAGACATC
cH78	H7	capture	TGGGARGGTYTSATYRAYGGRTGGTATGGYTTCAGRCATC
cH79	H7	capture	GGTTTAGCTTCGGGGCATCATGTTTTCTTCTTCTAGCCAT
cH710	H7	capture	GGTTTAGCTTCGGGGCATCATGYTTYMTWCTTCTDGCCAT
cH91	H9	capture	ATGGGATGCTRTGTGCAACAAAYCTGGGACRTCCYCTYAT
cH92	H9	capture	CAAGTGTGRCAACAGAAGATATAAATAGRACCTTCAAACC
cH93	H9	capture	GGGAGGTTGGTCAGGRYTAGTYGCTGGTTGGTATGGGTTC
cH94	H9	capture	TGAYCAGTGCATGGAGACAATTCGGAACGGGACCTAYAAC
cN791	N2†	capture	AAATCAGAAGATAATAACAATTGGCTCCGTCTCTCTAACC
cN792	N2†	capture	AAATCAGAAGATAATARCAATTGGYTCYGTYTCYCTAACY
cN793	N2†	capture	TGGGAACCAGACAAGTTTGCATAGCATGGTCCAGCTCAAG
cN794	N2†	capture	TGGSAACCARACAAGTKTGYATAGCATGGTCCAGCTCAAG
cN795	N2†	capture	GAAGTGCTCAGCATATAGAGGAATGTTCCTGTTATCCCCG
cN796	N2†	capture	GRAGTGCTCAGCATRTRGAGGAATGYTCCTGTTAYCCCCG
cN797	N2†	capture	ATCAATAGGTGTTTTTATGTGGAGTTAATAAGAGGAAGGC
cN798	N2†	capture	ATYAAYAGGTGTTTTTATGTRGAGTTRATAAGRGGAAGRC
cN31	N3	capture	TACCGAATTGCAGTGACACTATAATAACATACAATAATAC
cN32	N3	capture	CAGACTCCATTAAATCATGGAGAAAGGACATATTGAGAAC
cN33	N3	capture	TGGATGAGAATCAACAACGAGACTATACTGGAAACAGGGT
cN34	N3	capture	TAGTTACTTTCTGTGGATTAGACAATGAACCTGGATCGGG
pCtrl		capture	ACTGTTTGTTATCTTGTTATCGTTATCTGA
iFluBM1	Flu B M	intermediate	TGACAGAAGATGGAGAAGGCAAAGCAGAACTAGCAGAAAA*
iFluBM2	Flu B M	intermediate	TGAAGCATTTGAAATAGCAGAAGGCCATGAAAGCTCAGCG*
iFluBM3	Flu B M	intermediate	TCTCAGCTATGAACACAGCAAAAACAATGAATGGAATGGG*
iFluBM4	Flu B M	intermediate	GCTCTCCATTTCRTGGCTTGGACAATAGGRCATTTGAATC*
iFluBM5	Flu B M	intermediate	GAYCACATARTAATTGAGGGGCTTTCTGCYGAAGAGATAA*
iH21	H2	intermediate	CCARATATGYATYGGRTACCATKCCAATAATTCCACAGAG*
iH22	H2	intermediate	YTAYCCAGGCAGYTTCAATGATTATGARGAATTGAAACAT*
iH23	H2	intermediate	CAACTGGWGGTTCWCGGGCCTGYGCRGTRTCTGGYAAYCC*
iH24	H2	intermediate	YTTTCACAAYRTYCACCCAYTGACAATWGGTGAGTGCCCC*
iH25	H2	intermediate	ACAGCAATGAYCARGGATCAGGRTATGCAGCAGACAAAGA*
iH26	H2	intermediate	GGAAGAYGGRTTTCTWGATGTRTGGACATAYAATGCYGAR*
iH71	H7	intermediate	GGACCTCCCCAATGTGATCAATTCCTGGAGTTTTCCTCTG*
iH72	H7	intermediate	GGWCCWCCMCARTGYGAYCAATTYCTRGARTTTKMSKWTG*
iH73	H7	intermediate	AGTTGATAACAGTAAGAAGCTCAAAATACCAGCAATCATT*
iH74	H7	intermediate	AGTTGATAACAGTAGGAAGCTCGAAATACCAGCAATCATT*
iH75	H7	intermediate	AGYTRATAACAGTWGGRAGYTCBAADTAYCARCARTCHTT*
iH76	H7	intermediate	TCTGGCTACAGGAATGAGAAATGTTCCAGAGAAACCAAAG*
iH77	H7	intermediate	TTTGGCTACAGGAATGAGAAACGTCCCAGAGAACCCCAAG*
iH78	H7	intermediate	DYTGGCWACWGGRATGARRAAYGTYCCHGARAHYCCMAAR*

Oligo ID	Gene	Purpose	Sequences (5′ to 3′)
iH79	H7	intermediate	TTTGCATAAAGAATGGAAACATGCGGTGCACTATTTGTAT*
iH710	H7	intermediate	THTGYRTRAAGAATGGAAACATGCRGTGCACTATTTGTAT*
iH91	H9	intermediate	ATCGTYGAAAGACCATCGGCYGTTAATGGAWTGTGTTACC*
iH92	H9	intermediate	GTGGTAACTGTGYAGTGCAATGTCARACWGAAARAGGTGG*
iH93	H9	intermediate	GACATATGGRCWTATAAYGCAGAATTGCTAGTRCTGCTTG*
iH94	H9	intermediate	CTTACAAAATCCTCACCATTTATTCGACTGTCGCCTCATC*
iN791	N2	intermediate	TGGTGGAGACATTTGGATAACAAGAGAGCCTTATGTGTCG*
iN792	N2	intermediate	WGGTGGAGAYATYTGGRTRACAAGAGARCCTTATGTRTCR*
iN793	N2	intermediate	TGCATCAATGGGTCTTGTACAGTAGTAATGACTGATGGAA*
iN794	N2	intermediate	TGYATCAATGGRWCYTGTACRGTAGTAATGACKGATGGAA*
iN795	N2	intermediate	CAGGAGTGAAAGGATGGGCCTTTGACAGTGGAGATGATGT*
iN796	N2	intermediate	CAGGAGTGAAAGGGTGGGCCTTTGACAGTGGRRATGAYRT*
iN797	N2	intermediate	GCACTTATGGAACAGGCTCATGGCCTGATGGGGCGAACAT*
iN798	N2	intermediate	GYACYTATGGAACAGGCTCATGGCCTGATGGGGCGAAYAT*
iN31	N3	intermediate	GAATCCAAATCAGAAGATAATAACAATCGGGGTAGTGAAT*
iN32	N3	intermediate	GACAGAACACCATATAGGTCTCTGATCCGATTCCCAATAG*
iN33	N3	intermediate	CTTGTGTTGTTACTGTCACAGATGGCCCTGCTGCTAATAG*
iN34	N3	intermediate	ACACTGGTGTCCAACAATGATTGGTCAGGCTATTCAGGTA*
uni12‡	Flu A	RT	AGCAAAAGCAGG
uni13‡	Flu A	PCR	AGTAGAAACAAGG
unifluaf	Flu A	PCR	ACGACGGGCGACAAGCAAAAGCAGG
unifluar	Flu A	PCR	ACGACGGGCGACAAGTAGAAACAAGG
FluAMf	Flu A M	PCR	TCTAACCGAGGTCGAAACG
FluAMr	Flu A M	PCR	TGACAAAATGACCATCGT
FluBMf	Flu B M	PCR	TCGCTGTTTGGAGAC
FluBMr	Flu B M	PCR	TTTATTTGCTGACATTGATTAC
H22f	H2	PCR	AGCAAAAGCAGGGGTTAT
H22r	H2	PCR	AGTAGAAACAAGGGTG
N22f	N2	PCR	TCAGGGAGCAAAAGCAGGAG
N22r	N2	PCR	AGTAGAAACAAGGAGT
H7273f	H7	PCR	CATTCATTGCTTGTGTGCT
H7273r	H7	PCR	TCTCAAACTATATACAAAT
N72r	N2	PCR	ATAGGCATGAAATTGAT
N7292f	N2	PCR	CAGGAGTGAAAATGAATC
N22r	N2	PCR	AGTAGAAACAAGGAGT
H7273f	H7	PCR	CATTCATTGCTTGTGTGCT
H7273r	H7	PCR	TCTCAAACTATATACAAAT
N72r	N2	PCR	ATAGGCATGAAATTGAT
N7292f	N2	PCR	CAGGAGTGAAAATGAATC
N92r	N2	PCR	ATAGGCATGAAGTTGAT
N73f	N3	PCR	GAATCCAAATCAGAAGATAATA
N73r	N3	PCR	TACTTGGGCATAAACCCAAT
H92f	H9	PCR	GTTCCTGTGACACATGCCAAAG
H92r	H9	PCR	AAGGCAGCAAACCCCATT
N22f	N2	PCR	TCAGGGAGCAAAAGCAGGAG
N22r	N2	PCR	AGTAGAAACAAGGAGT

*25-mer poly (A) tail added at 3′ end of each intermediate oligonucleotide. H: hemagglutinin; N: neuraminidase; M: matrix; f: foward; r: reverse.
†Oligonucleotide designed from H7N2 and H9N2 subtypes.
‡Sequences from previous publications (*1,2*).

Technical Appendix Table 2. Summary of results from NGS data analysis for reference strains

Strains	NGS Total contigs/reads	Finding	Gene segment (length, bp) PB2 (2341)	PB1 (2341)	PA (2233)	HA (1778)	NP (1565)	NA (1413)	M (1027)	NS (890)
A/Puerto Rico/8/1934	8 / 122036	length (bp)	667	559	1552	1781	1210	1433	1028	1108
(H1N1)		read count	24	36	544	2056	6795	6549	196	51107
A/California/04/2009	7 / 93996	length (bp)	1846	2017	1827	1549	1555	754	1029	0
(pH1N1)		read count	3546	8335	3546	3996	9980	1246	18843	0
A/Japan/305/1957	8 / 60234	length (bp)	1658	1119	1341	1771	1206	1046	1114	882
(H2N2)		read count	7444	4253	3596	1621	4563	7302	2030	4253
A/Panama/2007/1999	4 / 106464	length (bp)	0	605	0	780	0	918	1026	0
(H3N2)		read count	0	423	0	130	0	178	8289	0
A/Vietnam/1203/2004	8 / 90962	length (bp)	2140	2205	2865	920	1580	1408	1042	697
(H5N1)		read count	8535	6103	14290	3170	14637	5537	18915	11715
A/turkey/Virginia/	8 / 63852	length (bp)	1780	1810	1671	1707	1570	1423	1031	886
4529/2002 (H7N2)		read count	632	334	2054	5978	8464	13057	9367	3440
A/RuddyTurnstone/	4 / 116382	length (bp)	0	509	0	0	1570	790	0	601
NJ/65/1985 (H7N3)		read count	0	6257	0	0	1267	4810	0	6257

Strains	NGS Total contigs/reads	Finding	Gene segment (length, bp) PB2 (2341)	PB1 (2341)	PA (2233)	HA (1778)	NP (1565)	NA (1413)	M (1027)	NS (890)
A/chicken/Hong Kong/G9/97 (H9N2)	7 / 65234	length (bp)	0	1756	1869	1729	1546	1458	939	690
		read count	0	6549	4963	6746	8456	5632	8908	4594
A/Minnesota/10/2012 (H3N2)	8 / 111564	length (bp)	1598	1925	2063	1099	1567	1470	1031	884
		read count	349	1816	4021	6847	19867	14500	21819	14762
A/Indiana/08/2011 (H3N2v)	8 / 66564	length (bp)	620	649	872	1762	1380	1054	1036	900
		read count	33	32	2572	2557	1931	1366	21325	13026
B/Brisbane/60/2008	7 / 57860	length (bp)	694	944	713	635	520	626	794	890
		read count	7011	10168	674	237	36	30	45	0

De novo assembly module was used in CLC Genomics Workbench software (v6.0.2) package for result handing to set parameter for mapping reads back to contiguous, similarity fraction, 0.9; length fraction, 0.5; mismatch cost 2; insertion cost 3 and deletion cost 3. Minimum contiguous length sets on 800 to assemble the consensus sequences and coverage is over 1000 reads. The sample subtype was verified using Influenza Research Database (IRD) for Identify Similar Sequences (ISS) (vBLASTN 2.2.22).

Technical Appendix Table 3. NGS data for detection and subtype of 24 clinical samples.

	NGS Detection				de novo assembling and bioinformatics analysis of segment (bp)								
Patient ID	Total contigs	Ave. reads	Flu A contigs	Ave. reads	PB2 (2341)	PB1 (2341	PA (2233)	HA (1778)	NP (1565)	NA (1413)	M (1027)	NS (890)	verified subtype
Flu001	8	216567	8	216567	2086	1922	2161	1771	1576	1479	1038	1086	H3N2
Flu002	8	233887	8	233887	2183	2204	2232	1771	1577	1613	1248	1060	H3N2
Flu004	7	208896	7	208896	2132	2017	1860	1895	1707	1817	1053	0	H3N2
Flu006	22	87902	8	203553	2302	1702	1870	1751	1769	1560	1208	1103	H3N2
Flu007	10	187419	8	232023	2123	1712	2060	1751	1576	1479	1251	891	H3N2
Flu008	20	50685	7	104105	2352	0	2371	1849	1620	1473	1037	945	H3N2
Flu009	8	85170	7	97168	1627	1754	2031	1764	1717	1693	1037	0	H3N2
Flu012	10	72106	7	72913	2105	2488	1791	1763	1575	1192	0	1068	H3N2
Flu013	8	194200	8	194200	1815	2071	2039	1762	1576	1297	1203	1121	H3N2
Flu014	9	243391	8	272331	2166	2447	1983	1828	1574	1484	1062	1042	H3N2
Flu017	10	95049	7	135026	2096	2572	2198	1764	1781	1680	0	977	H3N2
Flu018	7	76522	7	76522	1936	1957	1436	1705	1726	1901	0	891	H3N2
Flu021	8	119828	8	119828	2259	2351	2244	1952	1657	1594	1201	893	H3N2
Flu023	9	83396	8	93662	2196	2156	1929	1765	1574	1508	1325	1027	H3N2
Flu025	12	118080	8	170548	2349	2350	1990	1765	1572	1473	1036	899	H3N2
Flu026	12	155380	7	264292	1991	2475	2183	1909	1777	1557	1227	0	H3N2
Flu027	8	185997	8	185997	2352	2321	2071	1766	1579	1476	1650	893	H3N2
Flu028	8	163622	8	163622	2352	2348	2091	1765	1577	1475	1245	893	H3N2
Flu033	8	230527	8	230527	2244	2523	2390	1748	1631	1434	1243	892	H3N2
Flu034	12	132682	8	198210	2209	2011	2195	1761	1567	1467	1256	1115	H3N2
Flu036	8	229192	8	229192	2439	2611	1963	1881	1774	1677	1163	1077	H3N2
Flu037	8	120771	8	120771	2541	2153	2330	2094	1454	1476	1254	1231	H3N2
Flu038	10	103751	7	146822	2170	2091	2190	1959	1571	1473	1399	0	H3N2
Flu040	9	125152	7	160395	1976	1968	2062	1038	1776	1472	1035	0	H3N2

The sample subtype was verified using Influenza Research Database (IRD) for Identify Similar Sequences (ISS). All of the 181 genome sequences from 24 difference strains were submitted to the NCBI GenBank under accession nos. KJ741883–KJ742063 in April 2014.

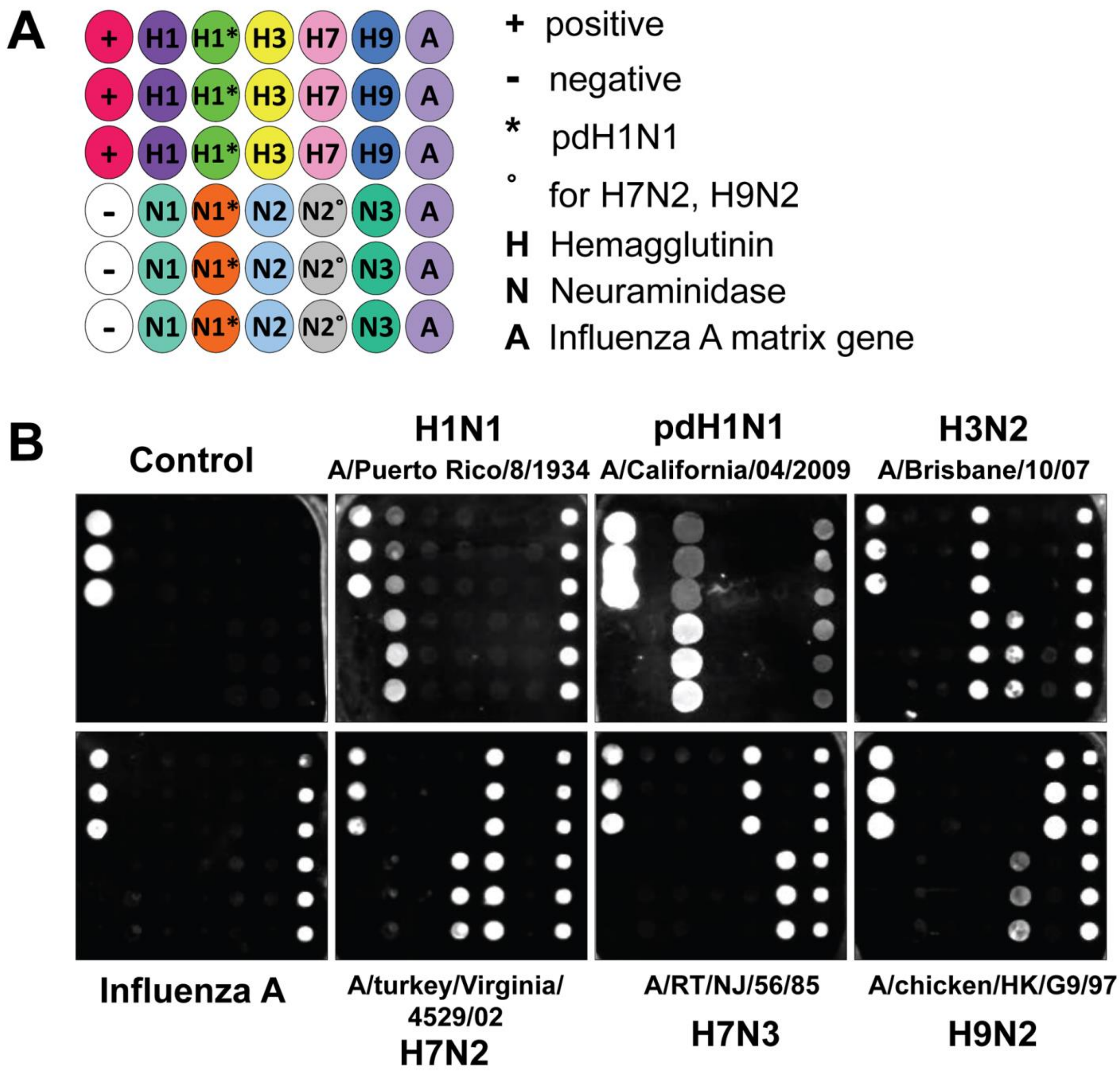

Technical Appendix Figure 1. Microarray layout (A) and sample image (B) for different subtypes of influenza A viruses. Positive control capture (closed red circles), negative control which uses printing buffer as capture (closed white circles), degenerated HA, NA and matrix gene captures (filled as variable color of closed circles) are indicated. A portion of the microarray images for DNA oligonucleotide following hybridization with PCR products are shown and light shades represent greater silver intensities for each genes. Typical nanomicroarray silver staining images represent the hits for specific subtypes indicated.

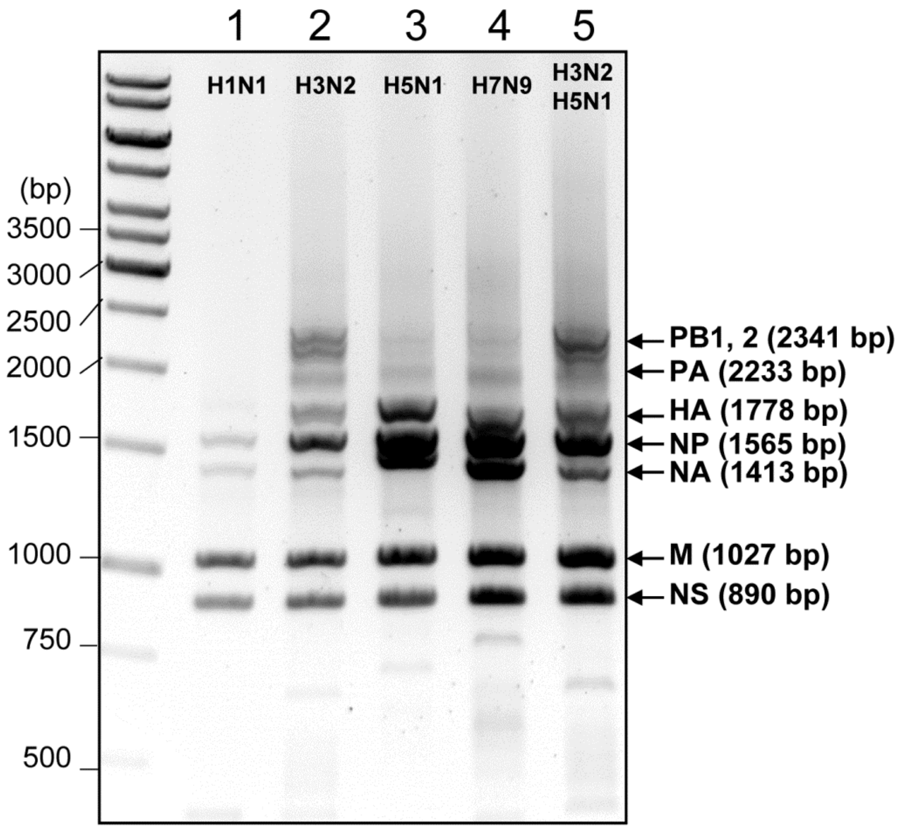

Technical Appendix Figure 2. Identification of whole genomic PCR amplicons from one or two mixed influenza A viruses. Viral RNA was extracted and RT-PCR was performed using adapted universal primer set . PCR products were electrophoresed on a 2% agarose gel. Lane 1: A/Puerto Rico/8/1934 (H1N1). Lane 2: A/Minnesota/10/2012 (H3N2). Lane 3: A/Vietnam/1203/2004 (H5N1). Lane 4: A/Anhui/1/2013 (H7N9). Lane 5: A/Minnesota/10/2012 (H3N2) and A/Vietnam/1203/2004 (H5N1).

References

1. Hoffmann E, Stech J, Guan Y, Webster RG, Perez DR. Universal primer set for the full-length amplification of all influenza A viruses. Arch Virol. 2001;146:2275–89. PubMed http://dx.doi.org/10.1007/s007050170002

2. Zhou B, Donnelly ME, Scholes DT, St George K, Hatta M, Kawaoka Y, et al. Single-reaction genomic amplification accelerates sequencing and vaccine production for classical and swine origin human influenza a viruses. J Virol. 2009;83:10309–13. PubMed http://dx.doi.org/10.1128/JVI.01109-09
